# Nonlinear fiber-bundle-cells-based phenomenological modeling of human tissue samples

**DOI:** 10.1007/s10237-022-01621-1

**Published:** 2022-10-26

**Authors:** László M. Vas, Péter Tamás, Eszter Bognár, Péter Nagy, Róbert Késmárszky, Károly Pap, Gábor Szebényi

**Affiliations:** 1 Department of Polymer Engineering, Faculty of Mechanical Engineering, Budapest University of Technology and Economics, 3 Műegyetem Rkp., Budapest, 1111 Hungary; 2 Department of Mechatronics, Optics and Engineering Informatics, Faculty of Mechanical Engineering, Budapest University of Technology and Economics, 3 Műegyetem Rkp., Budapest, 1111 Hungary; 3 IMEDIM Ltd., 18 Attila út, Budapest, 1013 Hungary; 4 FERR-VÁZ Ltd., 175 János u., Budapest, 1161 Hungary; 5 ONAKIA, Rond-point El Farouk, 7600 Kaweni, Mayotte, France; 6 Department of Orthopedics and Traumatology, Uzsoki Hospital, 29-41 Uzsoki Street, Budapest, 1145 Hungary

**Keywords:** Fiber bundle, Mechanical modeling, Load–strain curve, Human tissues, Failure map

## Abstract

Certain assemblies of fibers, called fiber bundles, play a crucial role in the statistical macroscale properties of fibrous structures like natural or artificial materials. Based on the concept of using idealized statistical fiber bundle cells (FBCs) as model elements, the software named FiberSpace was developed by us earlier for the phenomenological modeling of the tensile test process of real fibrous structures. The model fibers of these FBCs had been considered linear elastic, which was suitable for modeling certain textiles and composites. However, the biological tissues are multilevel structures with fiber-like building elements on every structural level where the fiber elements on the dominant level are statistical bundles of elementary fibers. Hence, their modeling required us to introduce model fibers of nonlinear mechanical behavior and derive the proper mathematical formulas for the calculation of the expected tensile force processes of the FBCs. Accordingly, we developed a new version of FiberSpace. The proposed nonlinear FBCs-based modeling method is essentially phenomenological that decomposes the measured and averaged stress–strain curve into the weighted sum of the responses of different idealized nonlinear FBCs. However, this decomposition can give certain information about the fibrous structure and some details of its damage and failure sub-processes. A special application of nonlinear E-bundles, where the measured stress–strain curve is expanded into a product-function series, may give another type of description for the failure process and can be applied to single measurements of structured failure process containing significant peaks and drops as well. The fitted phenomenological FBC models provide a decomposition of the measured force–strain curve, which enables to construct informative damage and failure maps. The applicability of the phenomenological modeling method and the fitting procedure is demonstrated with the tensile test data of some human and animal tissues, such as facial nerves and tendons.

## Introduction

Most human and animal tissues (e.g., tendon, muscle, bone, nerve, vein/artery, and skin or pellicle tissues) have a fibrous structure (Moore 1999; Neumann et al. 2019). Similar to artificial fibrous materials, such as textiles or fiber-reinforced composites (Bovier 2012; Fondrk et al. 1988; Gibson 2016; Hull 1996; Kollar 2003; Kovács and Romhány 2018; Takács and Szabó 2020; Vardai et al. 2019; Yin 2018), they are usually examined by using continuum phenomenological models (Fondrk et al. 1988; Nordin 2022; Pollintine et al. 2009) like the anisotropic linear elastic approach for hard materials and the isotropic Neo-Hookean and the anisotropic Fung and Holzapfel–Gasser–Ogden's (HGO) hyper-elastic models for soft materials (Huh et al. 2019; Sun and Sacks 2005). The mechanical features of fibrous materials, however, strongly depend on the statistical geometrical and mechanical properties of the building elements like fibrils, fibers, and bundles of them such as macro-fibrils, yarns or rovings, as well as on the connection between the fibers and their environment. Fiber bundles, especially the so-called classic type, have been studied since the first third of the last century and the results of Daniels and Jeffreys (1945), Harlow and Phoenix (1978), and Phoenix (Nato Advanced Study Institute on Mechanics of Flexible Fibre Assemblies), among others, have proved to be of fundamental importance. The classical fiber bundle models (FBMs) have rather widely been used in physics and in the brittle materials science (Hansen 2015; Nanjo 2016). These researchers focused mainly on strength as a probability factor. The deformation and damage behavior of fibrous structures during mechanical tests can be modeled with the so-called *fiber-bundle-cells* (FBC) method, where the FBC model is a network consisting of parallel and serial connections of statistical FBCs as model elements, which use linear elastic fibers (Molnar et al. 2012; Vas 2006a, 2006b; Vas and Rácz 2004; Vas et al. 2004; Vas and Tamás 2008). These fiber bundle cells represent different idealized and typified fiber properties such as fiber shape, state of deformation, connection to their environment (stiff or frictional grip), and the character of damage and the transmission of force. All the parameters determining the position, state, or strength of fibers are random variables. With the aid of the weighted parallel or serial connection of the fiber bundle cells, the mechanical behavior and the measured damage process of real fibrous systems can be modeled or identified from a fiber-bundle-cell model on the basis of measurements, and the structural properties of the systems can be determined.

A program package named FiberSpace was developed by the authors in Delphi Code earlier in order to help professionals construct a suitable linear FBC model for a given material or study the behavior of model structures where the model fibers were considered linear elastic (Molnar et al. 2012; Vas and Tamás 2008). Using FBCs for creating theoretical structural–mechanical models of fibrous structures provides a way to describe the statistical mechanical behavior of fibrous structures for different damage modes during mechanical tests such as tensile or bending tests.

Results of testing and analyzing woven fabric samples showed that instead of linear fiber elements, nonlinear elements had to be used for modeling tensile behavior (Vas et al. 2013). The use of such type of bundles can be found in some papers dealing with other structures as well (Goh et al. 2013; He and Wang 2004). We had similar results when testing biological (plant, animal, and human) tissues (Hangody et al. 2017). Hence, in order to model the tensile behavior of human or animal tissues, we developed the concept of nonlinear FBC modeling and a general approximation method based on nonlinear E-bundles in a new version of the FiberSpace software.

To demonstrate the applicability of the modeling method and this software, we modeled and evaluated tensile test results of human facial nerve and tendon samples, and analyzed them on the basis of some failure maps determined with the aid of the FBC model.

## Nonlinear fiber-bundle-cells-based modeling

### Nonlinear fiber bundle cells

**Fig. 1:**
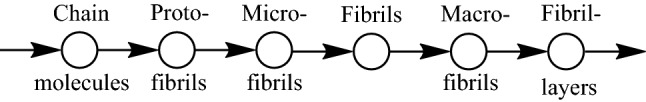
Partial structural graph of biological tissues

Studying the structure and mechanical properties of biological (plant, animal, and human) tissues has shown that they have a multilevel hierarchical structure where the fiber-like building elements, such as different types of fibrils are formed as the bundles of lower-level elements (e.g., a fibril is a bundle of the micro-fibrils) (Fig. 1) (Hangody et al. 2017; Hoagland 1997; Moore 1999; Nordin 2022). In addition, these lower-level elements may be crimped and/or oblique to a certain degree. Although their degree (micro-crimping and micro-obliquity) is much smaller than the degree of the higher level elements (macro-crimping and macro-obliquity), they may nevertheless strongly influence the mechanical behavior of the higher level elements. Therefore, even if the elementary fibers are linear elastic, the intermediate fibers, which are bundles of elementary fibers, cannot be modeled as linear, only nonlinear hyper-elastic materials.

Hence, in order to model the tensile behavior of animal and human tissues, we developed modified formulas to describe the expected tensile force process of the different nonlinear fiber bundles, and built these new formulas into a new version of the FiberSpace software.

#### Definitions of FBCs and the force–strain relationships of single fibers

**Fig. 2:**
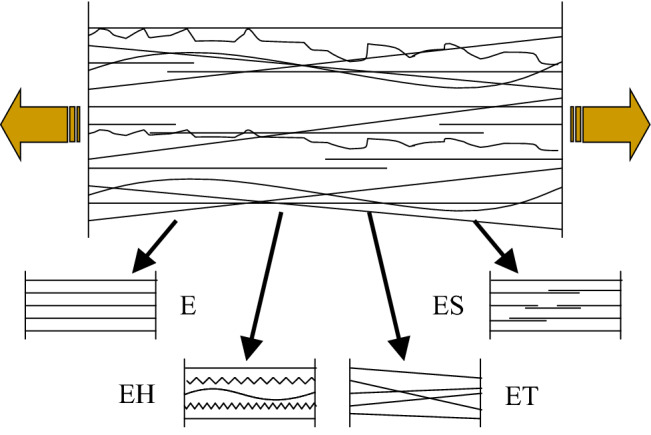
Classifying fibers into classes according to geometrical and mechanical properties and the structural schemes of the classes as idealized fiber bundle cells such as E-, EH-, ES-, and ET-bundles

Fibers in a fibrous structure that is subjected to a uniaxial tensile load (*F*) can be classified according to their geometry (shape, position) and mechanical behavior (strain state, gripping). These fiber classes are called fiber bundle cells (FBCs) (Vas and Rácz 2004; Vas and Tamás 2008) (Fig. 2). The model fibers are assumed to be elastic and perfectly flexible while they break at a random strain value. In an E-bundle (E is for elastic fibers) the fibers are straight and parallel to the load direction, they are not strained and are ideally gripped. The fibers in the other bundles have some statistical defect indicated by the second letter (Fig. 2). Therefore, H is a sign for the loose (crimped, wavy, or coiled) or pre-strained fibers. S is for the not ideally gripped fibers or fiber chains made of elementary fibers glued together at their ends hence they may slip out of the grips created by their vicinity. Finally, T is for the oblique or skew fibers characterized by the tangent of their orientation angle.

#### Strain of FBC fibers

Fibers of these FBCs are supposed to be perfectly flexible, nonlinearly elastic and to break at a random strain (*ε*_*B*_). In a constant-rate elongation tensile test, the strain (*ε*(*u*)[−]) and the tensile force (*F*(*u*)) of fibers create stochastic processes as a function of the bundle strain (*u*[−]). The formulas for fiber strain (*ε*) and the possible crosswise contraction (*W*(*u*)) of the oblique fibers (used in the case of linear FBCs as well) are as follows (Vas and Rácz 2004):

1 $$\varepsilon \left( u \right) = g\left( {u;\varepsilon_{0} ,T_{0} } \right) = \left( {1 + \varepsilon_{0} } \right)\sqrt {\frac{{\left( {1 + u} \right)^{2} + T_{0}^{2} W^{2} \left( u \right)}}{{1 + T_{0}^{2} }}} - 1$$

2 $$W\left( u \right) = \frac{1}{{\left( {1 + c_{a} u} \right)^{{c_{b} }} }}\sim 1 - c_{a} c_{b} u \left( {u \to 0} \right)$$

where *ε*_*0*_ is the possible initial strain (if *ε*_*0*_ > 0, the fiber is pre-strained, if *ε*_*0*_ < 0, the fiber is crimped), *T*_*0*_ = *tgα*_*0*_ is the initial obliquity of the fibers (*α*_*0*_ is the initial orientation angle) and *c*_*a*_*, c*_*b*_ are contraction constants. According to the asymptotic approximation in Eq. (2), the product of constant *c*_*a*_*c*_*b*_ can be understood as the Poisson’s coefficient of the fibrous system in the case of small deformations. For simplicity, all the stochastic parameters and variables are assumed to be independent.

#### Nonlinear tensile characteristics of FBC fibers

**Fig. 3:**
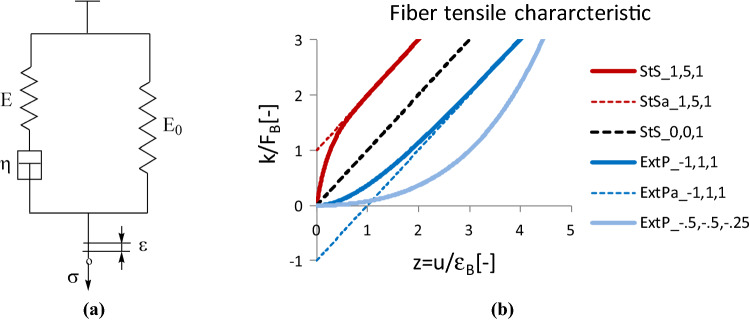
Standard-solid model (StS) (**a**) and the normalized theoretical shapes of possible responses to a stimulus of ramp type at different (**a**–**c**) parameters according to Eq. (4) (**b**)

We chose the response of a Standard-Solid model (Phenomenological Treatment of Viscoelasticity 2005; Vas et al. 2019) to a ramp-type stimulus as the nonlinear tensile characteristic function of the fibers (Fig. 3). This model is created by the parallel connection of a Maxwell branch and a spring—or its generalized version with several Maxwell branches (Vas et al. 2019). The model is often used to describe the mechanical behavior of hyper-elastic or viscoelastic materials such as elastomers (soft rubbers). The ramp-type stimulus is realized by the constant-rate elongation during tensile tests.

This engineering stress response function of the Standard-Solid model (Fig. 3a) is as follows:

3 $$\sigma \left( \varepsilon \right) = E_{0} \varepsilon + E\varepsilon_{0} \left( {1 - e^{{ - \frac{\varepsilon }{{\varepsilon_{0} }}}} } \right), \varepsilon_{0} = \dot{\varepsilon }_{0} \tau , \dot{\varepsilon }_{0} = \frac{v}{{l_{0} }}, \tau = \frac{\eta }{E}$$

where *E* > 0 and *E*_0_ > 0 are the elastic moduli of the springs, *η* > 0 is the dynamic viscosity of the viscous element, the dashpot, and *ε* ≥ 0 and *σ* ≥ 0, and $\dot{\varepsilon }_{0}$ are the engineering strain and stress, and the strain rate, respectively (Fig. 3a). *v* is the elongation rate, *l*_*0*_ is gauge length, and *τ* is the (relaxation) time constant of the Maxwell branch. As a formal generalization of Eq. (3), we denote the constants simply by *a*, *b*, and *c*. Allowing the negative values of *a* makes it possible to obtain not only a linear (*a* = 0) or a concave (*a* > 0) function shape but a convex (*a* < 0) shape as well (Fig. 3b). Moreover, when a large initial curved arc of the force–strain curve is to be modeled, the extension of the parameter domain for the negative *b* values can provide a simple way to describe it (Fig. 3b: ExtP; a—asymptote). However, it should be noted that in practical processes, the exponential rising is just the initial part of a logistic curve.

Consequently, as opposed to the one-parameter (*c*) linear tensile force–strain curves of the single fibers used earlier (Molnar et al. 2012; Vas and Rácz 2004; Vas and Tamás 2008), the nonlinear curves are described by the next 3-parameter (*a, b, c*) formula (Cleary 1979; Vas et al. 2013):

4 $$F_{f} \left( {\varepsilon \left( u \right)} \right) = A_{0} \sigma \left( {\varepsilon \left( u \right)} \right) = k\left( {\varepsilon \left( u \right)} \right) = c\varepsilon \left( u \right) + a\left( {1 - e^{ - b\varepsilon \left( u \right)} } \right)\sim \left\{ {\begin{array}{*{20}c} {\left( {c + ab} \right)\varepsilon \left( u \right), u \to 0} \\ {c\varepsilon \left( u \right) + a, u \to \infty , b > 0} \\ { - ae^{ - b\varepsilon \left( u \right)} , u \to \infty , b < 0} \\ \end{array} } \right.$$

where *k*(*ε*(*u*)) ≥ 0 and *A*_*0*_ is the cross-sectional area of the single fibers and *K*_*0*_ = *c* + *ab* is the initial tensile stiffness. In general, the parameters, *a*, *b*, and *c* are constant and may depend on the type of the FBC.

Obviously, this 3-parameter curve by Eq. (4) includes the linear one as well (*c* > 0, *a* = 0), moreover, when *b* > 0, it is asymptotically linear determined by slope *c* and intercepts *a*. If the fiber breaking strain distribution is known, the expected fiber breaking force can be calculated as follows:

5 $$\overline{F}_{B} = E\left( {F_{B} } \right) = E\left( {k\left( {\varepsilon_{B} } \right)} \right) = \overline{{k\left( {\varepsilon_{B} } \right)}} .$$

**Fig. 4:**
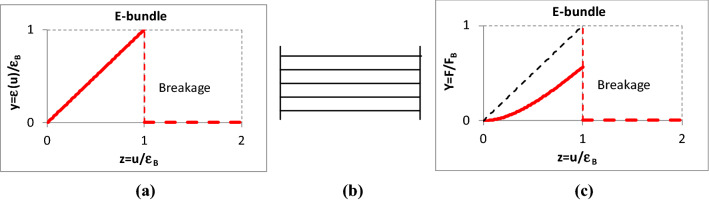
Relationship between the strain (**a**) and tensile force (**c**) of single nonlinear fibers and an E-bundle (**b**) strain

**Fig. 5:**
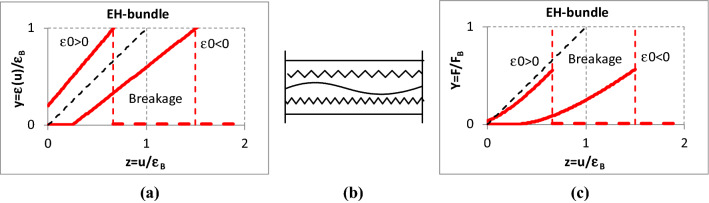
Relationship between the strain (**a**) and tensile force (**c**) of single nonlinear fibers and an EH-bundle (**b**) strain

**Fig. 6:**
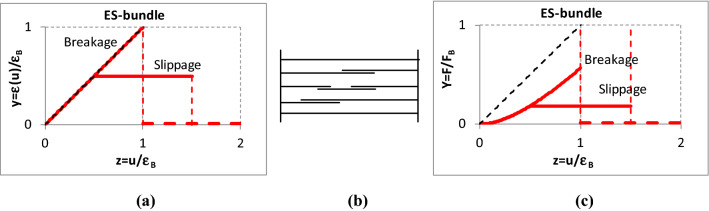
Relationship between the strain (**a**) and tensile force (**c**) of single nonlinear fibers and an ES-bundle (**b**) strain

**Fig. 7:**
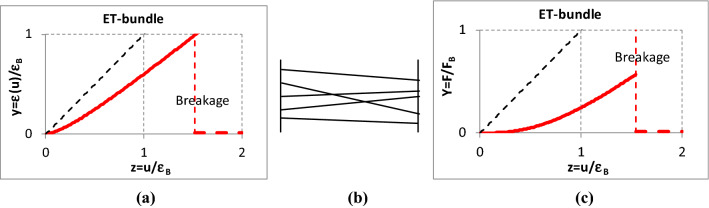
Relationship between the strain (**a**) and tensile force (**c**) of single nonlinear fibers and ET-bundle (**b**) strain

Besides the schematic of the bundles, Figs. 4, 5, 6, 7 show the normalized graphic relationships for the strain (*y* = *ε/ε*_*B*_) and the tensile force (*Y* = *F/F*_B_) of individual flexible and elastic fibers with both linear and nonlinear force–strain characteristics as a function of the bundle strain (*z* = *u/ε*_*B*_). The bundle strain-dependent fiber strain, *ε*(*u*), and the fiber force can be considered as stimulus and response, respectively. On the other hand, the relationship between the fiber and bundle strains, *ε*(*u*), represents and characterizes the mechanical connection between the single fiber and its material environment.

#### Tensile force response of the FBC fibers

The fibers of the *E-bundle* are straight and parallel to the load direction and they are ideally gripped, meaning that they do not slip out of the grips and do not break in the grips (Fig. 4). Therefore, it is often called ideal fiber bundle (Daniels and Jeffreys 1945; Harlow and Phoenix 1978; Nato Advanced Study Institute on Mechanics of Flexible Fibre Assemblies; Vas and Rácz 2004). As a consequence of its properties, the strain of each fiber (*ε*) in the E-bundle is equal to that of the bundle (*u*). Yet the overall relationship between the fiber and bundle strains is not linear if breakage is taken into account.

Vitiating the ideal properties of the E-bundle, one at a time, leads to three other bundle types which represent statistical behavior in some idealized way.

The fibers in the *EH-bundle* are ideally gripped but they may be loose (*ε*_*o*_ < 0) or pre-tensioned (*ε*_*o*_ > 0) with their chord remaining straight (Vas and Rácz 2004; Vas and Tamás 2008) (Fig. 5).

Fibers in the *ES-bundle* are straight and parallel but they may slip out of their grip at a strain level (*ε*_*S*_) or create fiber chains with slipping bonds (Fig. 6). The breaking strain-related slippage length is *ε*_*L*_ and the slippage ends at *ε*_*SL*_ = *ε*_*S*_ + *ε*_*L*_. The strain level of slippage is obviously the minimum of those obtained at the two finer ends as well as at the slipping bonds if there is any. Thus, the fibers in the ES-bundle can produce two types of failure depending on their stochastic parameters: slippage if *ε*_*S*_ < *ε*_*B*_ or breakage if *ε*_*B*_ < *ε*_*S*_. The slippage may model the flow in the fibrous structures.

The fibers are straight and ideally gripped but they may be oblique (the initial fiber angle, *α*_*0*_, is not zero, so *T*_*0*_ = *tgα*_*0*_ ≠ 0) in the *ET-bundle* (Fig. 7). The orientation angle of fibers may be a stochastic variable. In most cases, the expected value of the orientation angle is zero, meaning scattering about the load direction but a nonzero value means essential obliquity. The extreme case of the latter is when the orientation angle is a nonzero constant, modeling a kind of shearing.

Both the shape, position, and strength parameters of fibers are assumed to be independent stochastic variables. Consequently, the strain (*ε*(*u*)) and the tensile force (*F*(*u*)) of both the individual fibers and the bundles create multi-parameter stochastic processes as a function of the bundle strain (*u*).

#### Formulas for calculating the expected tensile force process of nonlinear FBCs

Knowing the relationship between the bundle (*u*) and fiber strains (*ε*), one can calculate the expected value of the tensile force of the FBCs ($E\left( F \right) = \overline{F}\left( u \right)$) as a sum of the single fiber forces, using the suitable formulas developed. Dividing the expected value by the mean breaking force of fibers, the normalized tensile force of the bundle is calculated as follows:

6 $$0 < FH\left( z \right) = \frac{{\overline{F}\left( {z\overline{\varepsilon }_{B} } \right)}}{{n\overline{F}_{B} }} = \frac{{\overline{F1} \left( {z\overline{\varepsilon }_{B} } \right)}}{{\overline{F}_{B} }} \le 1, z = \frac{u}{{\overline{\varepsilon }_{B} }}$$

where *n*, $\overline{F}_{B}$, and $\overline{\varepsilon }_{B}$ are the number, the mean breaking force, and the strain of fibers, respectively, and *z* is the bundle strain normalized by the mean breaking strain of fibers, while $\overline{F1}$ is the bundle force related to one fiber. Accordingly, normalizing the strain quantities in Eq. (1) with $\overline{\varepsilon }_{B}$ defines a new function:

7 $$h\left( {z;x,y} \right) = g\left( {z\overline{\varepsilon }_{B} ;x\overline{\varepsilon }_{B} ,y} \right) = \left( {1 + x\overline{\varepsilon }_{B} } \right)\sqrt {\frac{{\left( {1 + z\overline{\varepsilon }_{B} } \right)^{2} + y^{2} W^{2} \left( {xz_{B} } \right)}}{{1 + y^{2} }}} - 1.$$

Earlier we had developed mathematical formulas for calculating the expected value of the tensile force processes of linear fiber bundles (Vas and Rácz 2004) and built their numerical realization in the software named FiberSpace (Vas and Tamás 2008). Based on the nonlinear tensile characteristic of fibers, these mathematical relationships were modified. The related shape of the fiber strain by Eq. (7) and the normalized version of the formulas are presented subsequently for every FBC where *Q*_*X*_ is the distribution function of the stochastic variable *X* ∈ {*ε*_0_, *ε*_*S*_, *ε*_*SL*_, *ε*_*B*_, *T*_0_}.

### Nonlinear E-bundle

8 $$\varepsilon \left( u \right) = g\left( {u;0,0} \right) = u$$

9 $$FH\left( z \right) = \frac{{k\left( {z\overline{\varepsilon }_{B} } \right)}}{{\overline{{k\left( {\varepsilon_{B} } \right)}} }}\left( {1 - Q_{{\varepsilon_{B} }} \left( {z\overline{\varepsilon }_{B} } \right)} \right)$$

### Nonlinear EH-bundle

10 $$\varepsilon \left( u \right) = g\left( {u;\varepsilon_{0} ,0} \right) = \left( {1 + \varepsilon_{0} } \right)\left( {1 + u} \right) - 1$$

11 $$FH\left( z \right) = \frac{1}{{\overline{{k\left( {\varepsilon_{B} } \right)}} }}\mathop \smallint \limits_{{ - 1/\overline{\varepsilon }_{B} }}^{\infty } \left| {k\left( {y\left( {z,x} \right)} \right)} \right|_{ + } \left[ {1 - Q_{{\varepsilon_{B} }} \left( {y\left( {z,x} \right)} \right)} \right]dQ_{{\varepsilon_{0} }} \left( {x\overline{\varepsilon }_{B} } \right)$$

where *k*(0) = 0 and *k* is a strictly monotonically increasing function according to Eq. (4). Since the fibers are perfectly flexible, they cannot transmit negative (compressive) force, hence the positive part of the tensile characteristic is calculated from the positive part of fiber strain as well:

12 $$\left| {k\left( y \right)} \right|_{ + } = k\left( {\left| y \right|_{ + } } \right) = \left\{ {\begin{array}{*{20}c} {k\left( y \right),} & {y > 0} \\ {0,} & {y \le 0} \\ \end{array} } \right.$$

### Nonlinear ES-bundle

Equation (8) is valid for fiber strain in the ES-bundle as well.

13 $$FH\left( z \right) = \frac{{k\left( {z\overline{\varepsilon }_{B} } \right)}}{{\overline{{k\left( {\varepsilon_{B} } \right)}} }}\left( {1 - Q_{{\varepsilon_{B} }} \left( {z\overline{\varepsilon }_{B} } \right)} \right)\left[ {1 - Q_{{\varepsilon_{S} }} \left( {z\overline{\varepsilon }_{B} } \right)} \right] + \frac{1}{{\overline{{k\left( {\varepsilon_{B} } \right)}} }}\mathop \smallint \limits_{ - \infty }^{{z\overline{\varepsilon }_{B} }} k\left( {w\overline{\varepsilon }_{B} } \right)\left[ {1 - Q_{{\varepsilon_{B} }} \left( {w\overline{\varepsilon }_{B} } \right)} \right]\left[ {1 - Q_{{\varepsilon_{SL} }} \left( {z\overline{\varepsilon }_{B} - w\overline{\varepsilon }_{B} } \right)} \right]dQ_{{\varepsilon_{S} }} \left( {w\overline{\varepsilon }_{B} } \right).$$

### Nonlinear ET-bundle

The initial strain (*ε*_*0*_) is equal to zero, thus fiber strain is as follows:

14 $$\varepsilon \left( u \right) = g\left( {u;0,T_{0} } \right) = \sqrt {\frac{{\left( {1 + u} \right)^{2} + T_{0}^{2} W^{2} \left( u \right)}}{{1 + T_{0}^{2} }}} - 1.$$

The force components in the direction of the tensile load (*L*) and perpendicular to that (*T*) are given by

15 $$FH_{L} \left( z \right) = \frac{1}{{\overline{{k\left( {\varepsilon_{B} } \right)}} }}\mathop \smallint \limits_{ - \infty }^{\infty } \left| {k\left( {g\left( {z\overline{\varepsilon }_{B} ;0,x} \right)} \right)} \right|_{ + } \left[ {1 - Q_{{\varepsilon_{B} }} \left( {g\left( {z\overline{\varepsilon }_{B} ;0,x} \right)} \right)} \right]\frac{{\left( {1 + z\overline{\varepsilon }_{B} } \right)dQ_{{T_{0} }} \left( x \right)}}{{\sqrt {\left( {1 + z\overline{\varepsilon }_{B} } \right)^{2} + x^{2} W^{2} \left( {z\overline{\varepsilon }_{B} } \right)} }}$$

16 $$FH_{T} \left( z \right) = \frac{1}{{\overline{{k\left( {\varepsilon_{B} } \right)}} }}\mathop \smallint \limits_{ - \infty }^{\infty } \left| {k\left( {g\left( {z\overline{\varepsilon }_{B} ;0,x} \right)} \right)} \right|_{ + } \left[ {1 - Q_{{\varepsilon_{B} }} \left( {g\left( {z\overline{\varepsilon }_{B} ;0,x} \right)} \right)} \right]\frac{{xW\left( {z\overline{\varepsilon }_{B} } \right)dQ_{{T_{0} }} \left( x \right)}}{{\sqrt {\left( {1 + z\overline{\varepsilon }_{B} } \right)^{2} + x^{2} W^{2} \left( {z\overline{\varepsilon }_{B} } \right)} }}.$$

The limit of slippage resistance (*ε*_*S*_) and the slippage length (*ε*_*L*_) expressed as relative strains are independent of any other stochastic variables in the ES-bundle, which is well usable for studying different slipping or flow processes in general. However, when the fibers are for example molecule chains built in the crystalline part or micro-fibers of a short fiber-reinforced composite, slippage resistance and slippage length depend on the length of the fibers. In this case, instead of the ES-bundle, its modified versions, the ES1-bundle and the ES2-bundle can be used. In the ES1-bundle, the slipping resistance is constant during slippage, whereas it decreases linearly in the ES2-bundle. The linear ES1- and ES2-bundles were applied to describe the strength of fiber flows and unidirectional short fiber composites (Vas 2006a, 2006b). We plan to develop their nonlinear versions as a next step.

### Modeling and decomposing tensile test results with parallel-connected FBCs

We have developed an updated version of the program package named FiberSpace as the numerical realization of the nonlinear FBC-based modeling procedure, in order to assist the construction of a suitable FBC model for a given material or facilitate studying the behavior of model structures. The identification of the different statistical fiber bundle cells (FBCs) to be applied has been based on the minimization of the squared deviation between the measured force–strain curve and the expected tensile force process of the FBC model, which was created as the parallel connection of number-weighted fiber bundle cells.

#### Numerical realization of nonlinear FBCs by FiberSpace

**Fig. 8:**
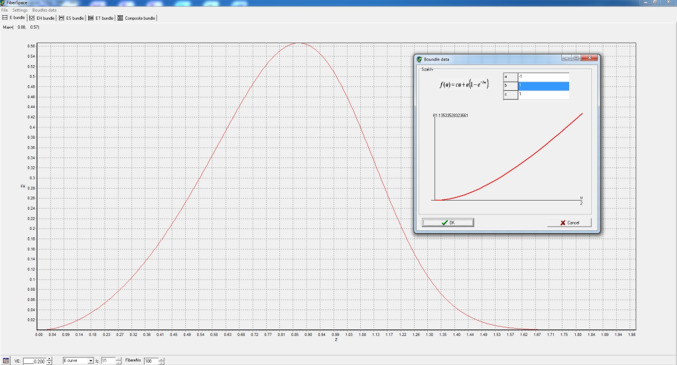
The tensile force process of a nonlinear E-bundle and the setting window for the nonlinear fiber characteristic as visible in the FiberSpace program screen

In Fig. 8, the normalized expected tensile force process of a nonlinear E-bundle can be seen together with the window for setting the tensile characteristic parameters of fibers as displayed by the novel version of FiberSpace.

The parameters (*a*, *b*, *c*) are the same as in Eq. (4). The window reveals the shape of the characteristic function as well. In addition, besides the exponential type characteristic by Eq. (4), FiberSpace offers two other modes for setting nonlinear fiber characteristics. One of them is a polynomial of a degree of maximum 5, the other is a numerical method where the measured data can be imported from MS Excel.

**Fig. 9:**
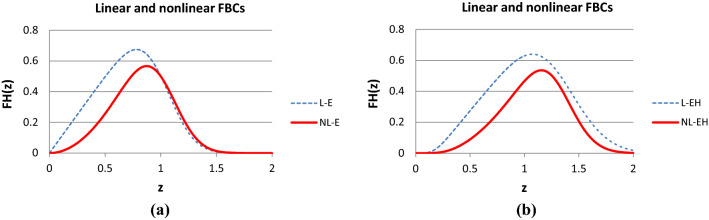
Normalized expected tensile force–strain curves of the E-bundle (AE = 1.0; VE = 0.2) (**a**) and EH-bundle (AE = 1.0, VE = 0.2; EH = −0.15 > 0 waviness, VH = 0.05) (**b**)

**Fig. 10:**
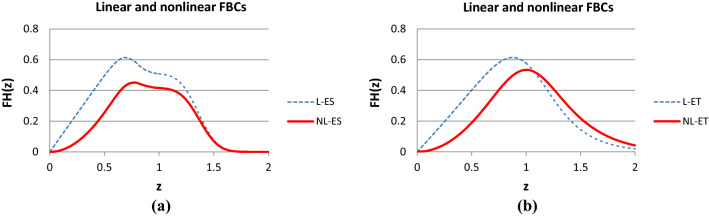
Normalized expected tensile force–strain curve of the ES-bundle (AE = 1.0, VE = 0.2; ES = 0.8, VS = 0.1, EL = 0.55, VL = 0.1) (**a**) and the ET-bundle (AE = 1.0, VE = 0.2; ET = 0.4, ST = 0.35, Ca = Cb = 0) (**b**)

Figures 9 and 10 show some typical results of calculating the normalized expected tensile process of the basic linear and nonlinear FBCs. They were calculated with the same model parameters except for those belonging to the fiber tensile characteristic. The parameters of the latter were *a* = *b* = 0 and *c* = 1 for the linear FBCs while they were *a* = -1 and *b* = *c* = 1 for the nonlinear FBCs. The diagrams were created with MS Excel. The short designations of the normalized expected value, E(X), and standard deviation, D(X), of the stochastic variables X ∈ {*ε*_*B*_, *ε*_*0*_, *ε*_*S*_, *ε*_*L*_, *T*_0_}, used in FiberSpace are the following: *AE* = E(*ε*_*B*_), VE = D(*ε*_*B*_)/AE; EH = E(*ε*_*0*_)/AE, VH = D(*ε*_*0*_)/AE; ES = E(*ε*_*S*_)/AE, VS = D(*ε*_*S*_)/AE, EL = E(*ε*_*L*_)/AE, VL = D(*ε*_*L*_)/AE; ET = E(*T*_0_), ST = D(*T*_0_), Ca = *c*_*a*_ and Cb = *c*_*b*_ are the contraction parameters, while the parameters of the nonlinear tensile characteristic of fibers are: Ya, Yb, Yc with the FBC code Y ∈ {E, EH, ES, ET}.

On the basis of Figs. 9 and 10, one can conclude that, relating to the linear E-bundle-curve (L-E), every stochastic disorder or damage decreases the expected tensile force values on the ascending parts and extends the range of the descending parts of the other linear bundles (blue curves for L-EH, L-ES, and L-ET). At the same time, the latter effect increases the mechanical reliability of the fiber bundles. On the other hand, in general, the introduction of nonlinear fiber characteristic decreases the tensile force values and modifies the shape of the initial ascending part of the curves while the descending part essentially remains similar to the linear case. In addition, the range of the descending part of the bundles NL-EH, NL-ES, and NL-ET is also wider related to that of the bundle NL-E. However, as can be seen in Fig. 9b, in the case of the EH-bundle, the slope of the descending part increases, which can compensate for the flattening effect of crimping and allows steeper descending. The latter can extend the possibilities of modeling tensile measurement results with FBCs.

#### The nonlinear FBC model—parallel-connected FBCs

**Fig. 11:**
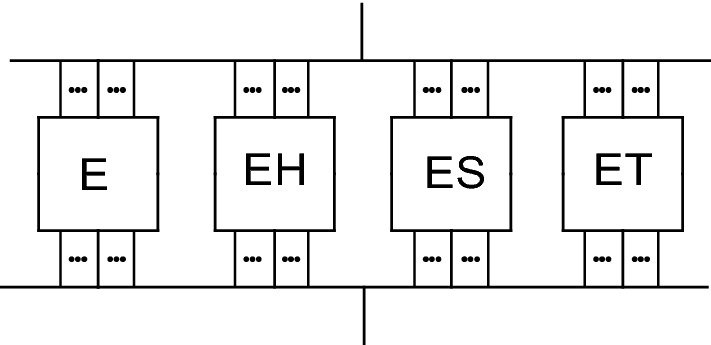
Parallel connection of the FBCs

The analysis of the bundle structure and the identification of the different statistical fiber bundle cells (FBCs) to be applied were based on the minimization of the squared deviation between the measured force–strain curve and the expected tensile force process of the FBC model, which was created as the parallel connection of number-weighted FBCs (Fig. 11). This latter model is called composite bundle.

The expected value process of a composite bundle (Fig. 11) is calculated as the weighted sum of the components (*w*_*k*_ is the weighting factor, that is, the fiber number ratio of the *k*-th FBC):

17 $$FH\left( z \right) = \mathop \sum \limits_{k = 1}^{4} w_{k} FH_{k} \left( z \right), \mathop \sum \limits_{k = 1}^{4} w_{k} = 1.$$

Weights can be given by arbitrary nonnegative integers *Se*, *Sh*, *Ss*, *St* for the E-, EH-, ES-, ET-bundles, respectively (their sum should be positive), and FiberSpace calculates the weighting fraction values *w*_*k*_, that is, the fiber number fractions, as follows, for example (*k* = 1 can be used instead of *k* = *e*):

18 $$w_{1} = w_{e} = Se/\left( {Se + Sh + Ss + St} \right).$$

**Fig. 12:**
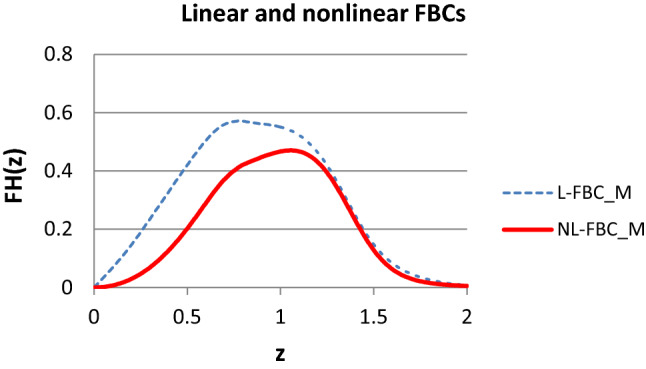
Expected tensile force process of parallel-connected linear (*a* = 0, *b* = 0, *c* = 1) and nonlinear (*a* = −1, *b* = 1, *c* = 1) FBCs with the weights *w*_*e*_ = 10%, *w*_*h*_ = 30%, *w*_*s*_ = 50%, *w*_*t*_ = 10%

Figure 12 shows the weighted sum of the normalized expected tensile force processes of the linear and nonlinear FBCs, which are shown in Figs. 9 and 10.

The weights used for the E-, EH-, ES-, and ET-bundles were 10%, 30%, 50%, and 10%, respectively.

The curves well demonstrate the possible effect of nonlinearity regarding the tensile characteristic of the fiber. Shifting to nonlinear behavior not only decreases the force values of the onset part of the curve but may change the shape as well. Among others, the slightly descending plateau of the ES-bundle becomes slightly ascending.

The load–strain curve of a real fibrous sample is determined with a tensile test and its normalized form can be imported into FiberSpace from Excel files with the aid of the Microsoft Component Object Model technology.

By default, FiberSpace treats the *z* values between 0 and 2 with an adjustable partition. In the partitioning points, the normalized load–strain curve is calculated by interpolation on the basis of the measured strain values. The measured load-deformation data or the normalized result of modeling can be normalized or denormalized, respectively. The 16-model parameters used in the linear case, such as the 4-component weights and the data of the 12 statistical bundles are completed with a maximum of 12 nonlinearity parameters of the different bundle types (the maximum is for the case when all the FBCs have different tensile fiber characteristics). Consequently, including the mean breaking force of the fibers ($\overline{F}_{B}$) used for normalization, which may be unknown as well, the functional (*Ψ*) to be minimized in order to obtain the structural and mechanical data of the FBC model is of 29 parameters represented by vector *p*. The best approximation of a measured load-deformation curve *F*(*u*) can be determined with a fitting procedure based on the least squares method:

19 $$\Psi \left( {\underline {p} } \right) = \mathop \sum \limits_{i = 0}^{N} \left( {\frac{{F\left( {z_{i} \cdot A} \right)}}{B} - FH\left( {z_{i} ,\underline {p} } \right)} \right)^{2} \to \min !$$

where *Z*_*0*_ is the limit of the normalized strain domain, and A = AE, B = $n\overline{F}_{B}$ are the normalizing factors of the measured force–strain curve.

#### Fourier approximation and the final model parameters

Since the FBC model may use up to 29 parameters, determining them by minimizing the deviation according to Eq. (19) needs suitable initial parameter values. The measured and normalized load–strain curve is approximated by Fourier regression so that its shape features can be recognized. On the basis of our experiences, the load–strain curves can be well approximated with a Fourier polynomial of 8 terms besides the constant.

The FiberSpace system uses the gradient method for seeking the global minimum which depends on the initial values of the model parameters. In order to find the adequate initial position, we built in the so-called nearest neighbor searching system. The already determined Fourier coefficient vectors (*f* = [*a*_0_, *a*_1_,…, *a*_*n*_, *b*_1_,…, *b*_*n*_]^T^) and the related parameter vectors (*m* ∈ **R**^*P*^) containing the *P* = 29 FBC model parameters are stored in database Data. The input data of the deducing system are formed by the Fourier coefficient (*f*) determined from the normalized measured tensile force–strain curve, FN(*z*), while the output data are the initial parameters ($\underline{{\hat{p}}}$) of the model composite bundle. The final model parameters (*p*) are obtained by minimizing the expression according to Eq. (21):

20 $$FN\left( z \right)\to ^{ Fourier } \underline {f} \to ^{Deduction} \underline{{\hat{p}}} \to ^{ FH } FH\left( {z,\underline{{\hat{p}}} } \right)\to ^{Minimizing} FH\left( {z,\underline {p} } \right) \approx FN\left( z \right).$$

Finally, when *d*_1_ > 0, the vector pair (*f*, *p*) is stored in data as one record of the training data. This constitutes a step of machine learning.

### Modeling with a series of nonlinear E-bundles

Using the FBCs introduced and discussed above leads to a decomposition of the measured tensile force–strain relationship. This gives information on the fiber classes of the fibrous structure tested and represents stochastic structural imperfections, such as wavy, crimped, oblique, pre-stressed fibers, or the possibility of fiber pullout. At the same time, the application of nonlinear E-bundles only may be very advantageous when the purpose of decomposition is to determine sub-bundles corresponding to certain known conditions (e.g., damage/failure modes, elements of hierarchic structural levels).

On the other hand, the mechanical behavior of the nonlinear EH-, ES-, and ET-bundles can be decomposed into weighted parallel combinations of nonlinear E-bundles therefore the expected responses of the previous FBCs can be decomposed into the weighted sum of the nonlinear E-bundle-responses (*j* ∈ {E,EH,ES,ET}):

21 $$FH_{j} \left( u \right) \approx \mathop \sum \limits_{i = 1}^{{n_{j} }} w_{ji} FH_{ji} \left( u \right) = \mathop \sum \limits_{i = 1}^{{n_{j} }} w_{ji} k_{ji} \left( u \right)R_{ji} \left( u \right)$$

where the reliability functions are the complement distribution functions of the fiber breaking strain, which are of the same two parameter type with different expected values (*m*_*i*_ = E(*ε*_*Bi*_)) and standard deviations (*s*_*i*_ = D(*ε*_*Bi*_)).

**Fig. 13:**
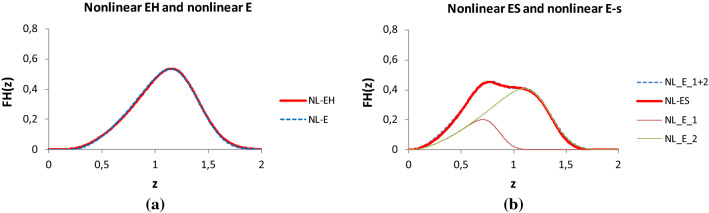
Approximating the normalized tensile response of a nonlinear EH-bundle (see Fig. 9b) (**a**) and ES-bundle (see Fig. 10a) (**b**) with parallel-connected nonlinear E-bundles. **a** a shifted E-bundle (*a* = −1, *b* = 1.5, *c* = 1.5, *u*_0_ = 0.2, *m* = 0.67, *s* = 0.2; RMSE = 1.04%, *R*^2^ = 0.999). **b** Two E-bundles (*u*_0_ = 0; *w1* = 0.4; *a1* = −0.8, *b1* = 2.125, *c1* = 1.7, *m1* = 0.83, *s1* = 0.11; *w2* = 0.6, *a2* = −0.55, *b2* = 2.09, *c2* = 1.15; *m* = 1.3, *s2* = 0.17; RMSE = 1.67%, i^2^ = 0.998)

In general, in order to show and analyze the effects of statistical inhomogeneities in the structure, the fibers in different linear or nonlinear FBCs have the same mechanical properties but according to Eq. (21), the fibers in a series of E-bundles have different tensile characteristics. This fact makes further analysis possible. Figure 13 shows the approximation of normalized force responses of nonlinear EH- and ES-bundles with a single and two nonlinear E-bundles, respectively. Using the simplest way, the large initial arc of the nonlinear EH-response was approximated by a shifted E-bundle response (Fig. 13.a; *b* > 0; *FH*_*E*_(*u-u*_*0*_) > 0 if *u* > *u*_*0*_ and = 0 if *u*_*0*_), however, the exponential rising (*b* < 0) could be used as well.

Uniting the different approximations, the measured tensile force–strain curve, *F*(*u*), can be modeled by the weighted parallel connection of nonlinear E-bundles (*n* = Σ*n*_*j*_), hence it can be approximated with the expected tensile process of the resultant bundle:

22 $$F\left( u \right) \approx \overline{f}\left( u \right) = \mathop \sum \limits_{i = 1}^{n} w_{i} \overline{f}_{i} \left( u \right) = \mathop \sum \limits_{i = 1}^{n} w_{i} k_{i} \left( u \right)R_{i} \left( u \right), \mathop \sum \limits_{i = 1}^{n} w_{i} = 1.$$

On the other hand, Eq. (22) represents the law of total expectation, namely:

23 $$F\left( u \right) \approx \overline{f}\left( u \right) = \mathop \sum \limits_{i = 1}^{n} E\left( {f\left( u \right){|}B_{i} } \right)P\left( {B_{i} } \right) = \mathop \sum \limits_{i = 1}^{n} k_{i} \left( u \right)R_{i} \left( u \right)w_{i} \le \mathop \sum \limits_{i = 1}^{n} k_{i} \left( u \right)w_{i} = \overline{k}\left( u \right)$$

where $\overline{k}\left( u \right)$ is the expected tensile characteristic, hence, the conditional expected value of *f*(*u*) and the probability of the condition are given by:

24 $$E\left( {f\left( u \right){|}B_{i} } \right) = k_{i} \left( u \right)R_{i} \left( u \right), P\left( {B_{i} } \right) = w_{i} ,$$

respectively, where *B*_*i*_ is the event that an arbitrary fiber belongs to the *i*^th^ sub-bundle.

It should be noted that Eq. (22) is a finite part of a special function series of product-shaped terms thus the decomposition into nonlinear E-bundles is equivalent to an expansion into series (bundle-expansion). Moreover, taking into consideration that the values *m*_*i*_ (*i* = 1,..,*n*) create a strictly monotonically increasing series

25 $$m_{1} < m_{2} < \ldots < m_{n}$$

the sum according to Eq. (22) can be converted into an integral form as follows:

26 $$F\left( u \right) \approx \overline{f}\left( u \right) = \mathop \smallint \limits_{0}^{\infty } k\left( {u;m} \right)R\left( {u;m} \right){\text{d}}W\left( m \right)$$

where *W*(*m*) = P(*μ* < *m*) is the distribution function of *μ,* which is the expected fiber breaking strain as a conditional expectancy. Taking into consideration that the tensile characteristic, *k*(*u*), can be given by the relaxation spectrum, which characterizes the damage-less work of the material, one can suppose that the density function *w*(*m*) = d*W*/d*m* and the *s*(*m*) standard deviation function can be regarded as damage parameter spectra characterizing the failure process.

Here, *s*^2^(*m*) is a conditional variance of *μ*, thus it should obviously contain the variance of *μ* therefore the next expression may be suitable to use:

27 $$s\left( m \right) = \sqrt {s_{0}^{2} + \omega^{2} \left( {m - M} \right)^{2} } , E\left( {s^{2} \left( \mu \right)} \right) = s_{0}^{2} + \omega^{2} E\left[ {\left( {m - M} \right)^{2} } \right] = s_{0}^{2} + \omega^{2} S^{2}$$

where *s*_*0*_ and *ω* are constants, and *M* = E(*μ*) and *S* = D(*μ*) are the expected value and the standard deviation of *μ*. This can be decomposed into a sum of such variances:

28 $$s^{2} \left( m \right) = \mathop \sum \limits_{i = 1}^{n} \left[ {s_{0i}^{2} + \omega_{i}^{2} \left( {m - M_{i} } \right)^{2} } \right] = \mathop \sum \limits_{i = 1}^{n} s_{0i}^{2} + \mathop \sum \limits_{i = 1}^{n} \omega_{i}^{2} \left( {m - M_{i} } \right)^{2} .$$

Note that the sum is equivalent to a polynomial of 2nd order like in Eq. (27).

The relaxation spectrum determines the deformation behavior in the undamaged state, while the damage parameter spectra characterize the failure process. Fitting the model by Eq. (22) and determining the free parameters (*a*_*i*_*, b*_*i*_*, c*_*i*_*, m*_*i*_*, s*_*i*_)(*i* = 1,…,*n*) are performed by minimizing the squared deviation according to Eq. (19), from the measured data. Taking into consideration that the series in Eq. (22) can be estimated from above by a kind of Prony-series that can be characterized by a so-called discrete relaxation spectrum (Hoagland 1997), {(*b*_*i*_; *a*_*i*_*, c*_*i*_)}, which consists of double values; besides that, the original series itself can be a damage or failure parameter spectrum, {(*b*_*i*_; *m*_*i*_*, s*_*i*_)}, which contains double values as well. It can be easily seen that *b*_*i*_ is a kind of “strain-frequency” or relaxation parameter since it can be expressed with the relaxation time, *τ*_*i*_:

29 $$\frac{1}{{b_{i} }} = \dot{u}_{0} \tau_{i} = \dot{u}_{0} \frac{{\eta_{i} }}{{E_{i} }}$$

where $\dot{u}_{0}$ is the engineering strain rate ($u = \dot{u}_{0} t$), and *η*_*i*_ and *E*_*i*_ are the dynamic viscosity and the tensile modulus, respectively, which are the parameters of the Maxwell model used for the tensile characteristic of the model fibers (Fig. 3a).

The nonlinear E-bundles, as the simplest FBCs, can be calculated even in an MS Excel environment, and we used them first for modeling and analyzing the results of tensile and acoustic emission tests (Vas et al. 2019).

### FBC model-based damage maps and reliability characteristics

The FBC model fitted to tensile test measurements makes it possible to calculate some qualitative characteristics of the material, such as the reliability characteristics and damage maps of different weighting.

According to Eq. (17), the resultant normalized expected tensile force of the FBC model is the weighted sum of those of the components. The gradual addition of the components decomposes the resultant curve into ranges which show the weighted fraction of the components and the represented damage modes as a function of the normalized FBC strain (*n* = 1,…,*N* ≤ 4):

30 $$FH\left( z \right)_{n} = \mathop \sum \limits_{i = 1}^{n} w_{i} FH_{i} \left( z \right)$$

where *N* is the number of components with nonzero weight. The normalized form of Eq. (30) is this:

31 $$fH\left( z \right)_{n} = \frac{{\mathop \sum \nolimits_{i = 1}^{n} w_{i} FH_{i} \left( z \right)}}{{\mathop \sum \nolimits_{i = 1}^{N} w_{i} FH_{i} \left( z \right)}}$$

According to Eq. (9), the normalized expected tensile characteristic of the E-bundle, *kH*_*E*_ (*z*), is identical with that of the fibers, consequently, the reliability characteristic equals the complement distribution function of the fiber breaking strain:

32 $$kH_{E} \left( z \right) = \frac{{k\left( {z\overline{\varepsilon }_{B} } \right)}}{{\overline{{k\left( {\varepsilon_{B} } \right)}} }}, RH_{E} \left( z \right) = 1 - Q_{{\varepsilon_{B} }} \left( {z\overline{\varepsilon }_{B} } \right).$$

The normalized expected tensile characteristic of the other FBCs can be obtained by substituting the complement distribution function of the damage variables (*ε*_*B*_, *ε*_*S*_, and *ε*_*BL*_) with the unit-step function in Eqs. (11), (13), and (15). For the ES-bundle, this leads to the same function as that of the E-bundle while for the EH- and ET-bundles, we obtain Eq. (33) and (34), respectively:

33 $$kH_{EH} \left( z \right) = \frac{1}{{\overline{{k\left( {\varepsilon_{B} } \right)}} }}\mathop \smallint \limits_{{ - 1/\overline{\varepsilon }_{B} }}^{\infty } \left| {k\left( {y\left( {z,x} \right)} \right)} \right|_{ + } {\text{d}}Q_{{\varepsilon_{0} }} \left( {x\overline{\varepsilon }_{B} } \right)$$

34 $$kH_{ET,L} \left( z \right) = \frac{1}{{\overline{{k\left( {\varepsilon_{B} } \right)}} }}\mathop \smallint \limits_{ - \infty }^{\infty } \left| {k\left( {g\left( {z\overline{\varepsilon }_{B} ;0,x} \right)} \right)} \right|_{ + } \frac{{1 + z\overline{\varepsilon }_{B} }}{{\sqrt {\left( {1 + z\overline{\varepsilon }_{B} } \right)^{2} + x^{2} W^{2} \left( {z\overline{\varepsilon }_{B} } \right)} }}{\text{d}}Q_{{T_{0} }} \left( x \right)$$

In this case, *RH*_*E*_(*z*) gives the fraction of the fibers intact at the strain considered, hence *RH*_*E*_(*z*) is the reliability function of the E-bundle.

The reliability characteristic of the *i*th component, *RH*_*i*_(*z*), can be obtained when its expected tensile force, *FH*_*i*_(*z*), is divided by the related expected tensile characteristic, *kH*_*i*_(*z*), which represents the failureless behavior of the bundle at any strain load:

35 $$RH_{i} \left( z \right) = \frac{{FH_{i} \left( z \right)}}{{kH_{i} \left( z \right)}}$$

Accordingly, in a weighted sense, the reliability characteristic has a similar role concerning the damage and failure of fibers.

The reliability characteristic of the *i*th component, *RH*_*i*_(*z*), can be obtained when its expected tensile force, *FH*_*i*_(*z*), is divided by the related expected tensile characteristic, *kH*_*i*_(*z*), which represents the failureless behavior of the bundle at any strain load:

36 $$RH\left( z \right) = \frac{FH\left( z \right)}{{kH\left( z \right)}} = \mathop \sum \limits_{i = 1}^{N} w_{i} \frac{{FH_{i} \left( z \right)}}{kH\left( z \right)} = \mathop \sum \limits_{i = 1}^{N} \frac{{w_{i} kH_{i} \left( z \right)}}{kH\left( z \right)}RH_{i} \left( z \right)$$

where *kH*(*z*) is the resultant expected tensile characteristic of the FBC model:

37 $$kH\left( z \right) = \mathop \sum \limits_{i = 1}^{N} w_{i} kH_{i} \left( z \right).$$

Similarly to Eq. (30), a kind of damage or failure map can be constructed by adding the component reliability characteristics together gradually (*n* = 1,..,*N*):

38 $$RH\left( z \right)_{n} = \mathop \sum \limits_{i = 1}^{n} w_{i} \frac{{FH_{i} \left( z \right)}}{kH\left( z \right)}.$$

In the case of modeling the tensile force-extension measurements directly, the suitable formulas can be obtained by denormalizing Eqs. (30)-(38).

### Determining the normalizing parameters

In general, the aim of normalizing measured or modeled data is to transform them into a dimensionless coordinate system, reasonably into the interval [0,1] as it is for the tensile force in FBC modeling:

39 $$0 \le FH\left( u \right) = \frac{F\left( u \right)}{{F_{N} }} < 1; 0 < z = \frac{u}{{u_{N} }}$$

where *F*_*N*_ and *u*_*N*_ are the normalizing parameters that are suitable for the measured force (*F*) and strain (*u*) values, respectively.

In the ideal case, the mean breaking force ($\overline{F}_{B}$) and strain ($\overline{\varepsilon }_{B}$) of fibers are known from single fiber tensile measurements, and if the total number of the fibers (*N*) is known as well, normalization can be performed like in Eq. (9):

40 $$F_{N} = N \cdot \overline{F}_{B} ;\quad u_{N} = \overline{\varepsilon }_{B} .$$

In FiberSpace, these are denoted by AE (= *u*_*N*_) and B (= *F*_*N*_) (see, e.g., Fig. 12).

When the sample to be modeled is a fibrous system of unknown structure and it is built up of unknown fibers as building elements, the normalizing parameters should be estimated from the measured tensile force–strain curve. The latter can often be approximated with a non-normalized (linear or nonlinear) E-bundle relationship similar to Eq. (9), as follows:

41 $$F\left( u \right) \approx k\left( u \right)\left( {1 - Q_{{\varepsilon_{N} }} \left( u \right)} \right)$$

where *k*(*u*) is the tensile characteristic fitted to the ascending part of the curve and *Q* is the distribution function of a virtual “fiber breaking strain” (*ε*_*N*_). If *ε*_*N*_ is of symmetric distribution such as normal distribution, then at the expected value of *ε*_*N*_ ($u = \overline{\varepsilon }_{N}$), *Q* equals ½. This means that when the FBC model consists of an E-bundle, only then can *u*_*N*_ = $\overline{\varepsilon }_{N}$ and *F*_*N*_ = *k*($\overline{\varepsilon }_{N}$) be used as normalizing parameters. Moreover, if the relative SD of *ε*_*N*_ is small enough, *F*_N_ = *k*($\overline{\varepsilon }_{N}$) may be a correct estimation. In other cases, let us denote the coordinates of the intersection point of *k*(*u*)/2 and *F*(*u*) with (*u*_1_, *F*(*u*_1_)), and the coordinates of the (global or first) force peak with (u*, F* = *F*(u*)):

42 $$u_{1} : \frac{1}{2}k\left( {u_{1} } \right) = F\left( {u_{1} } \right), u^{*} :\mathop {\max }\limits_{u} F\left( u \right) = F\left( {u^{*} } \right).$$

These coordinates determine some lower and upper bounds for the normalizing parameters:

43 $$F^{*} < k\left( {u^{*} } \right) < F_{N} = k\left( {u_{N} } \right) \le k\left( {u_{1} } \right); u^{*} < u_{N} \le u_{1} .$$

## Application to tensile test results of human tissues

In order to demonstrate the applicability of the modeling and evaluation methods, samples of human facial nerves and tendons were tested, modeled, and analyzed.

### Materials and tensile test results

#### Human facial nerve samples

**Fig. 14:**
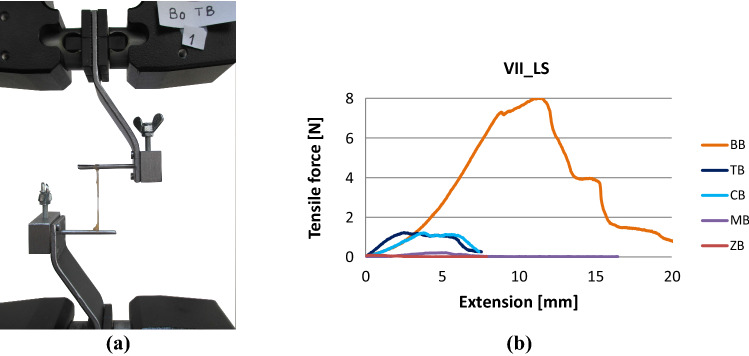
Arrangement of the tensile test of human facial nerves (**a**) and the results (**b**)

The tensile tests were performed with a Zwick Z005 computer-controlled tensile tester with custom grips, where the nerves were rigidly fixed on steel slit rods. Test speed was 10 mm/min. In Fig. 14a, the arrangement of the tensile test of some facial nerves (Table 1) of a 59-year-old man (Fig. 14a) can be seen, while Fig. 14b shows the results. Table 2 contains the geometrical and mechanical data of the specimens.

**Table 1:** Identity codes of facial nerves tested

Code	Explanation
VII	Nervus facialis
VII_LS_TB	VII, left side, temporal branch
VII_LS_ZB	VII, left side, zygomatic branches
VII_LS_BB	VII, left side, buccal branches
VII_LS_MB	VII, left side, mandibular branches
VII_LS_CB	VII, left side, cervical branch

**Table 2:** Geometrical and tensile test data of the left side facial nerves (nervus facialis)

Type of nerve	Short code	*L* [mm]	*l*_0_ [mm]	*l*_1_ [mm]	*t*_0_ [mm]	*T*_0_ [mm]	*t*_1_ [mm]	*T*_1_ [mm]	*F*_max_ [N]
Left side VII_LS	TB	32	8	17	2.90	4.50	2.31	2.42	1.23
	ZB	24	4	24	1.46	2.69	1.06	1.11	0.06
	BB	48	24	38	1.37	2.64	0.65	0.77	8.01
	MB	40	11	37	1.93	3.17	0.98	2.07	0.23
	CB	34	6	12	0.98	1.70	0.62	0.68	1.21

The force–elongation curves in Fig. 14.b are significantly different, hence averaging them point by point would not give a reasonably usable result for modeling or analyzing. Therefore, both their evaluation and modeling are to be performed on the individual measurements.

For modeling and analysis, the facial nerve sample Code BB was selected because of its large bent initial part convex from below and structured failure process containing interesting peaks and drops.

#### Tendon samples

Before tensile testing, the prepared human cadaver tendon samples (harvested within a maximum of 24 h post mortem, stored at −80 °C in a radio-protectant solution and subjected to 42 kGy dose virucidal gamma irradiation) were subjected firstly to a static load of 50 N for 30 s and after that, a fatigue load of 2000 tensile cycles. The applied waveform was sinusoidal, with a force between 50 and 250 N, and at a frequency of 2 Hz.

The tensile testing of the samples was performed on an INSTRON 8872 servo-hydraulic tester (maximum load 25 kN, gripping mode: freezing jaws (Hangody et al. 2017)) with a constant extension rate of 20 mm/min. Before the test, the samples were pre-tensioned by a tensile load of 150 N at this rate, in order to eliminate possible gripping errors.

**Fig. 15:**
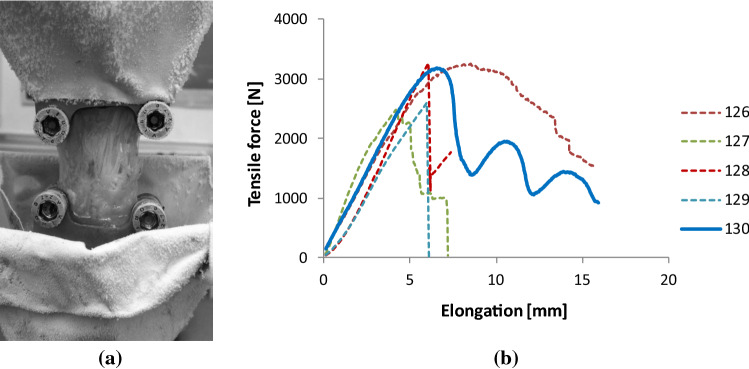
Arrangement of the tensile test of tendons (**a**) and the results (**b**)

In Fig. 15, the arrangement of testing and the graphical results of the tensile test of some tendons after fatigue are depicted while the numerical data can be found in Table 3. Similarly, as above, the tensile force–elongation curves are significantly different, thus it is reasonable to carry out modeling on single measurements.

**Table 3:** Geometrical and tensile test data of the human tendons tested (Semit. + Grac. = Semitendinosus + Gracilis, ant. = anterior, Perosn. long. = Peroneus longus)

Code	Type	Width [mm]	Thickness [mm]	Gauge length [mm]	*F*_max_ [N]	Elong. at *F*_max_ [mm]	Tensile stiffness [N/mm]	Breaking elong. [mm]
126	Quadriceps	15	2	50	3251	8.5	564	15.8
127	Semit. + Grac	9	3		2487	4.2	830	7.2
128	Tibialis ant	9	3		3268	6.1	654	7.4
129	Perosn. long	9	3		2598	6.0	536	6.1
130	Achilles	16	2		3179	6.6	562	15.9

For modeling and analysis, tendon sample Code 130 was selected because of its specially structured failure process showing three large separated peaks.

### FBC modeling of the test results

Two modeling methods were used based on fiber bundles. One included parallel-connected nonlinear FBCs of different types (E, EH, ES, and ET) and the other was the parallel connection of a series of nonlinear E-bundles. In modeling, the elongation or strain at break was assumed to be of normal distribution. The goodness of approximation was characterized by the absolute squared error (denoted as Difference in FiberSpace) or the relative mean squared error (RMSE) and the determination coefficient (R^2^). RMSE is the square root of mean difference related to the maximum measured force at the given scaling, while R^2^ is the squared value of the linear correlation coefficient between the modeled and measured force values.

#### Modeling the tensile behavior of a human facial nerve

##### Decomposition into nonlinear FBCs

As mentioned above, the tensile test result of the left side facial nerve code BB was modeled and analyzed with the aid of the FiberSpace software.

**Fig. 16:**
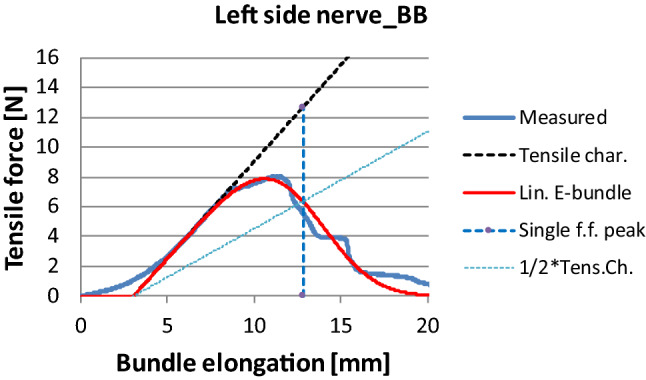
Tensile force vs. bundle strain curve of the facial nerve modeled by shifted linear (**a**) and nonlinear (**b**) E-bundles (Tensile char.—Tensile characteristic, Lin.—linear, Single f.f. peak—mean fiber force peak value obtained from single fiber tensile tests, ½*Tens.Ch.—straight line with half a slope related to the Tensile char.)

The first step before creating an FBC model is the normalization of the measured force–elongation curve, which needs the normalizing parameters according to Eq. (39). On the basis of Paragraph 2.5, for example, they can be estimated by fitting a simple shifted linear E-bundle to the measured data, as shown in Fig. 16, where the tensile characteristic was approximated by the inflection tangent of the rising part (its Equation is *y* = 4.65 + 1.3(*x*−6.61)). From fitting the linear E-bundle (*a* = 0), we obtained that E(*ε*_*B*_)≈*ε*_*N*_ = 12.80 mm and the tangent gave at this place that E(*F*_*B*_)≈*F*_*N*_ = 12.69 N, hence the shifting of the E-bundle response came at about 3.03 mm.

**Fig. 17:**
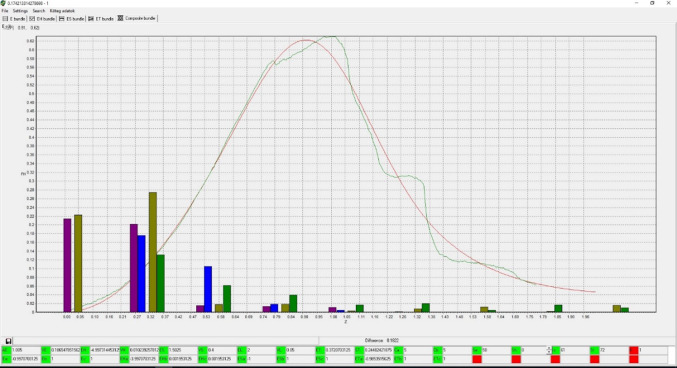
The result of seeking the best approximation by FiberSpace: the normalized measured (green) and modeled (red) curves as visible in the FiberSpace program screen

Figure 17 shows the final approximation (red) of the measured and normalized force–elongation relationship (green) obtained by FBC modeling in the FiberSpace environment, together with the sine and cosine Fourier amplitudes as parameters (olive and green columns of the measured sample, and purple and blue columns of the approximating FBC), which were stored in the learning process of the software. The goodness properties of approximation were RMSE = 3.39% and *R*^2^ = 0.988.

**Fig. 18:**
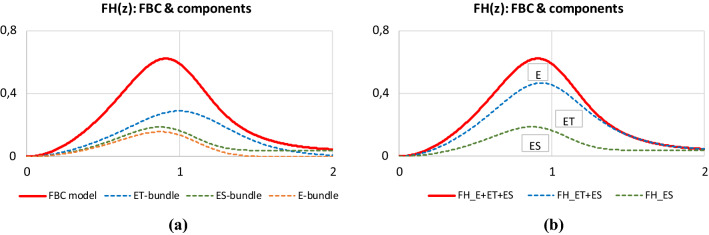
Normalized results of modeling the facial nerve BB: FBC components (**a**), and the map obtained by adding them (**b**)

Figure 18 shows the component curves belonging to FBCs and the parameters are summarized in Table 4. The tensile characteristic parameters of all the fibers were the same (*a* = −1, *b* = 1, *c* = 1) while ET contraction parameters were Ca = Cb = 5, meaning a very strong contraction by the reduction of the free volume among fibers. An ideal normalization leads to AE = 1.0. Here, a bit of correction was carried out by FiberSpace (Table 4). In this case, the fiber tensile characteristics found by fitting have a large initial curved part (*b* < 0), hence the EH-bundle, which models the effect of the crimped or loose fibers, was not needed, therefore FiberSpace canceled it by giving it zero weight. According to the results in Table 4, only about 27% of the fibrils creating the facial nerve behave like well-aligned and ideally gripped fibers (E-bundle), while 73% can be considered oblique (ET-bundle) or not ideally gripped (ES-bundle) fibers.

**Table 4:** Parameters of the nonlinear FBC model of the facial nerve BB

FBC code	*w*%	AE	VE	ES	VS	EL	VL	ET	ST
E	27.3	1.01	0.19						
ES	33.3			1.50	0.40	2.00	0.05		
ET	39.4							0.37	0.25

Moreover, mean obliquity is not zero (ET = 0.37), meaning that the oblique fibers may be aligned following a kind of spiral. Regarding the ES-fibers, both the mean slippage threshold (ES = 1.5) and the mean slippage length (EL = 2.0) are high, hence the majority of this type of fibers break.

**Fig. 19:**
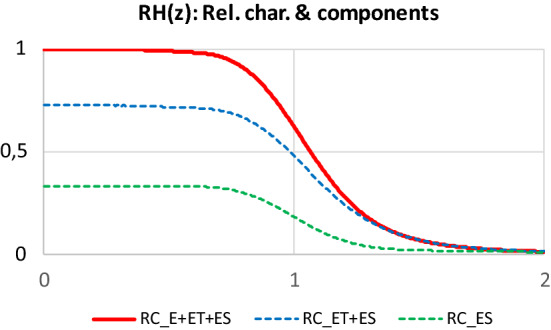
Reliability characteristic of the facial nerve BB and its components

In Fig. 18a, the weighted component curves can be seen, while in Fig. 18b, their sum gives a map regarding their participation in the resultant resistance force (calculated with Eq. (30)). Figure 19 shows the reliability map of the components and their sum computed with Eq. (38), which shows a division similar to that in Fig. 18b.

On the basis of Fig. 18, it can be stated that the fraction (27, 33, 40%) and role of the FBCs as sub-bundles created by ideal (E), oblique (ET), and slipping (ES) fibers in the force response are essentially similar considering the resistance force and so is their role regarding reliability (Fig. 19). Yet, the overall significant role of the oblique fibers should be stressed, except for very large deformations, where the slipping fibers of the ES-bundle dominate mechanical behavior.

##### Decomposition into a series of nonlinear E-bundles

**Fig. 20:**
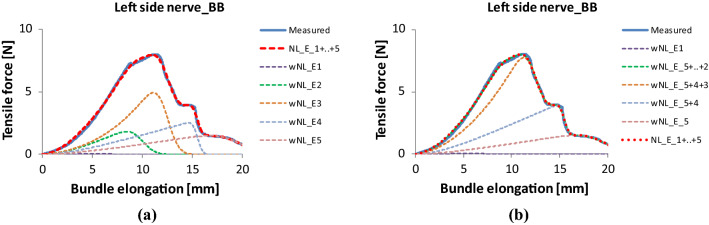
Modeling of the facial nerve Code BB with a series of E-bundles: the weighted component responses (**a**), and the map obtained by adding them (**b**)

According to Fig. 17, the FBC model curve gives a rather good mean profile, but it does not follow the strongly structured failure process of such a single measurement. Based on the parallel connection of a series of E-bundles, the force response of which is given by Eqs. (9) and (41) in a simple product form, the measured force–elongation curve can be approximated with the sum of the component responses corresponding to Eq. (30) (Fig. 20a). All this results in a decomposition of the measured profile into small parts as well as the area below the measured curve into “slices” bounded by the tensile characteristics of the E-bundle components (Fig. 20b). Hence, this operation is a kind of slicing and layering decomposition, where sectioning and layering can follow both the different arcs in the rising part and the peaks and drops in the falling part of the measured single curve.

In this case, decomposition leads to five E-bundle components as can be seen in Fig. 20. Their parameters are summarized in Table 5. Because of the better approximation, the goodness properties improved significantly (RMSE = 1.64%, *R*^2^ = 0.9974) compared to those of the general FBC model.

**Table 5:** Parameters of the E-bundle components

Component code	w [%]	A [N]	b [1/mm]	c [N/mm]	K_0_ [N/mm]	m [mm]	s [mm]	V [%]
1	3.0	−1.78	0.325	0.58	0	6.0	2.0	33.3
2	30.0	−3.69	0.325	1.20	0	9.8	1.0	10.2
3	41.0	−14.47	−0.095	−1.25	0.13	12.3	0.9	7.3
4	17.0	−4.00	0.325	1.30	0	15.5	0.4	2.6
5	9.0	−4.00	0.325	1.30	0	19.5	1.8	9.2

Analyzing the data in Table 5 reveals that the values of parameter b are positive in four cases but it is negative in the case of component 3, meaning a steep exponential tensile characteristic. In the latter case, the tensile characteristic of the model fibers is significantly exponential therefore the model parameter “c” cannot be interpreted as asymptotic stiffness. Moreover, this bundle is dominant because it describes the largest drop in force, thus it has the maximum weight (41%). The use of this kind of bundle is necessary since the initial part of the measured curve has a large curved part (convex from below) and the E-bundles cannot describe that with the fiber tensile characteristic given by Eq. (4) if parameter b is positive, in contrast to the general FBC model, which can include an EH-bundle containing wavy or crimped fibers.

**Fig. 21:**
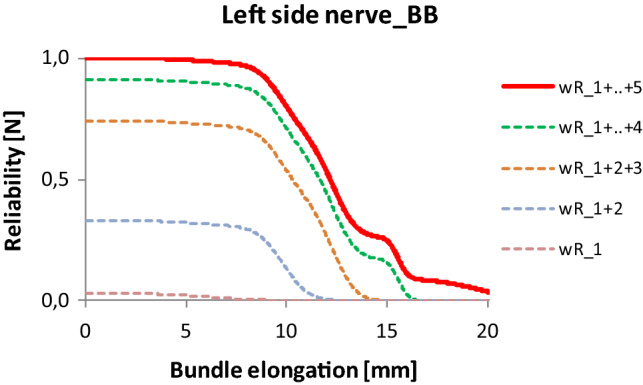
Reliability of the E-bundle series model of facial nerve Code BB

Comparing the component and resultant reliability of the general FBC model (Fig. 19) and the E-bundle series model (Fig. 21) shows that the significant difference can be observed mainly at larger deformations, where the latter can follow the structured failure process measured.

Comparing the results of general FBC modeling with those obtained by E-bundle modeling, one can conclude that the former gives a kind of internal description regarding the structural inhomogeneities, while the latter provides a boundary decomposition.

#### Modeling the tensile behavior of tendons after fatigue

**Fig. 22:**
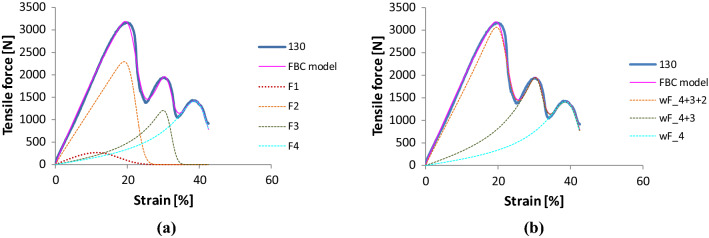
Modeling the tendon Code 130 with a series of E-bundles

The measured force–elongation curve of the tendon Code 130 has three large bulky peaks, hence we modeled that with the parallel connection of four E-bundles (Fig. 22, Table 6). The good approximation is characterized by RMSE = 2.63% and *R*^2^ = 0.9885.

**Table 6:** Parameters of the nonlinear E-bundles

Component code	w [%]	a [N]	b [1/%]	c [N/%]	K0 [N/%]	m [%]	s [%]	V [%]
1	16.0	26.5	1.00	28.4	54.9	15.0	5.0	33.3
2	70.0	26.0	1.00	124.4	150.4	22.2	1.8	8.1
3	7.6	−13.5	−0.14	13.5	15.4	31.9	1.3	4.1
4	6.4	−11.4	−0.12	11.4	12.7	41.5	2.4	5.8

Three of the components describe the peaks while component 1 models the initial damage and failure events, the scattering of which is the largest (33% in Table 6). The dominant component is number 2 belonging to the largest peak, hence its weight is 70%. The visible part of the two smaller peaks needed large curved tensile characteristics (Fig. 22b) consequently the *b* values of the corresponding components 3 and 4 became negative (Table 6).

**Fig. 23:**
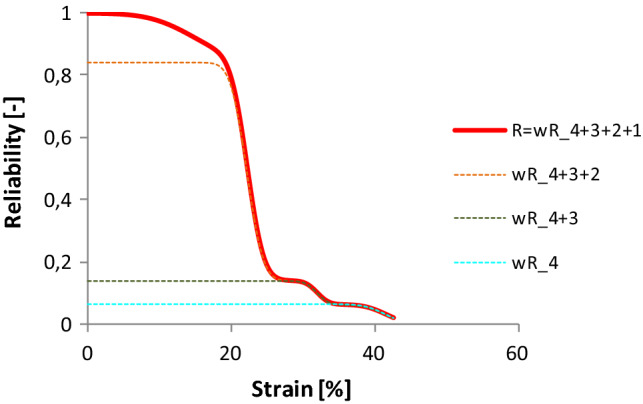
The reliability function of tendon Code 130 and its components

**Fig. 24:**
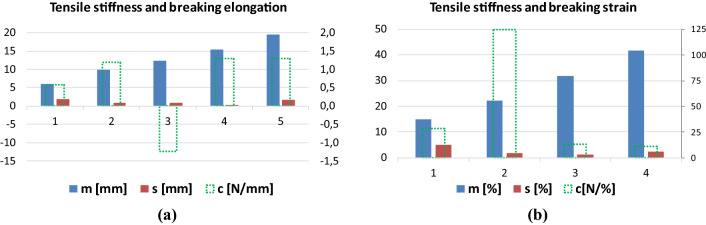
Tensile stiffness parameters (*c*) and the mean (m) and the standard deviation (s) of the breaking elongation [mm] or the strain [%] parameters of the E-bundle components for facial nerve BB (**a**) and tendon Code 130 (**b**)

Similar relations can be observed regarding component reliabilities (Figs. 23, 24). The dominance of component 2 is obvious, while at the same time the last two peaks provide a certain reliability over 25% strain.

#### Damage spectra of the failure process

The work of viscoelastic structural elements under load without damage or failure can be characterized with the so-called relaxation spectrum (*τ*), which is a relationship between relaxation time (a sort of reaction time), and the elastic modulus of parallel-connected Maxwell models. This is a kind of density function with a domain of [0,∞).

Based on the concept established by Eqs. (22)–(29), E-bundle series modeling makes it possible to characterize the damage and failure process of the samples tested, and also the reliability maps, by some relationships of spectrum type that may be called damage spectra. In this case, the mean breaking elongation or strain (*m*) can play the role of relaxation time according to Eqs. (22) and (26), while the dependent variable may be the weight (*w*) or the standard deviation of the breaking strain (*s*). Nevertheless, it must be taken into account that, according to Eqs. (23) and (24), all these are based on the conditional expected values and the law of total expectation applied to the tensile force process.

**Fig. 25:**
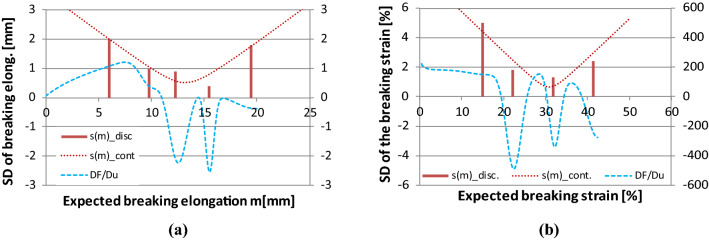
Discrete and continuous damage spectra of facial nerve BB (*M* = 13.22 mm, *s*_*0*_ = 0.51 mm, *ω*^2^ = 0.0723) (**a**) and tendon Code 130 (*M* = 31 mm, *s*_0_ = 0.63 mm, *ω*^2^ = 0.075) (**b**)

**Fig. 26:**
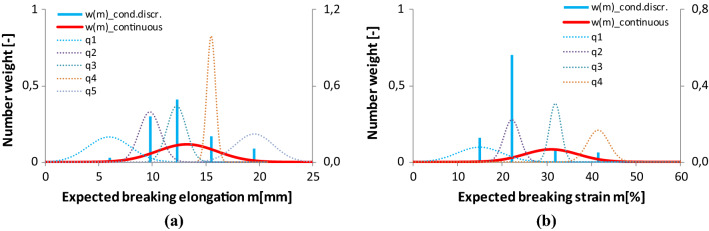
Component weights as well as conditional and resultant number density functions of the fiber breaking strain for facial nerve BB (M = 13.22 mm, *S* = 2.8 mm) (**a**) and tendon Code 130 (*M* = 31%, *S* = 6%) (**b**)

For the sake of comparison, Figs. 24, 25, 26 show together the graphical results of the damage analysis of both human facial nerve BB and tendon Code 130.

The column diagrams in Fig. 24 show the asymptotic tensile stiffness (*c*) of the fibers characterizing the intact work of the E-bundle components, as well as two damage properties, the mean values (*m*) and the standard deviation (*s*) of fiber breaking elongation or strain. The absolute values of facial nerve stiffness (*c*) increase monotonically (Fig. 24a and Table 5) with *m* as opposed to those of the tendon, where the second one is much larger than the others, highlighting its dominance in the tensile force (Fig. 24b). In both cases, the mean breaking elongation or strain increases almost linearly confirming its applicability as independent spectrum variable, while the medium values of standard deviation are smaller than those at the edges (Fig. 24a, b).

The latter refers to the form according to the quadratic formula by Eq. (27). Indeed, in Fig. 25a, b, the relationship between the mean values and the standard deviation can be seen where the measured values create a discrete line spectrum, which is approximated by a continuous function, *s*(*m*), which can be determined by fitting the formula by Eq. (27). The minimum point of *s*(*m*) belongs to the overall mean value of fiber breaking strain (*M*). The goodness of fitting was essentially satisfactory (facial nerve: RMSE = 11.6%, *R*^2^ = 0.85; tendon: RMSE = 12.2%, *R*^2^ = 0.82) considering the small amount of data.

Also, it can be observed in Fig. 25 that the *m* values can be found about the minimum places or the inflection point of the derivative of the resultant model tensile force (DF/Du), meaning that they characterize the places of intensive damage. The smaller the standard deviation (SD) values are, the steeper changes or drops in the tensile force are modeled by the E-bundle component in question. This can be observed as well when comparing the component density functions of the breaking elongation or strain depicted with dotted lines in Fig. 26 to the SD values in Fig. 25. These are non-weighted conditional probability density functions that are considered normal with parameters N(*m*, *s*^2^) and the component weights are indicated by the blue columns. Besides them, in Fig. 26, the resultant non-conditional density function, *w*(*m*), is plotted with a solid red line, which is assumed to be normal with parameters N(*M*,*S*^2^). The expected value, *M*, is the same as in Fig. 25, but the standard deviation, S, was estimated to satisfy the next condition related to the mean breaking strain values of the component (*m*_*i*_):

44 $$M - 3S < \mathop {\min }\limits_{i} m_{i} < \mathop {\max }\limits_{i} m_{i} < M + 3S.$$

The *S* value estimated in this way can be made more accurate by fitting the integral form according to Eq. (26) to the measured force–elongation relationship.

## Conclusions

The idealized statistical nonlinear fiber-bundle-cells (FBCs) and the modeling methods developed are suitable for phenomenological modeling of multilevel fibrous structures such as human tissues and make it possible to analyze them on the basis of evaluating measurements and determining the ratio of fibers or their bundles of different geometrical properties. The software FiberSpace uses composite bundles constructed as the parallel connection of different nonlinear FBCs for modeling the tensile load–strain curve of real fibrous structures. The expert system based on Fourier approximation and load–strain curve classes makes it possible to find the best model parameters fast. The system can learn and develop itself by improving mapping and/or establishing new subclasses on the basis of the modeled results.

The two modeling methods developed can be applied as tools in different problems. The use of different FBCs with the same or different fiber tensile characteristics is based on average mechanical behavior, since in this case, the expected tensile processes are calculated. In this way, the FBC model can reveal some structural details related to statistical inhomogeneities, such as wavy and oblique fibers or weaker connections between fibers and their environment, and the effects of all these problems on mechanical behavior.

However, when a tensile load–strain curve is the result of a single measurement, its modeling and analysis may be performed more advantageously by decomposition based on a series of nonlinear E-bundles.

The biomechanical applicability of both modeling methods was shown by using them for evaluating and analyzing the tensile test measurements performed on some human tendons and facial nerves.

The proposed FBC modeling method is related to the evaluation of tensile tests and valid for elastic model fibers, however, it can be extended to bending, shearing, and torsion tests and for viscoelastic model fibers as well. The presented results may give a suitable basis for further development regarding the application of the statistical FBC model as material law in the finite element simulation of fibrous structures of real geometry, subjected to real mechanical load which is the long-range aim of this work.
